# An electromechanical model of neuronal dynamics using Hamilton's principle

**DOI:** 10.3389/fncel.2015.00271

**Published:** 2015-07-16

**Authors:** Corina S. Drapaca

**Affiliations:** Department of Engineering Science and Mechanics, Pennsylvania State UniversityUniversity Park, PA, USA

**Keywords:** electromechanics, dynamic stiffness, Kelvin–Voight model, Hodgkin–Huxley model, Hamilton's principle

## Abstract

Damage of the brain may be caused by mechanical loads such as penetration, blunt force, shock loading from blast, and by chemical imbalances due to neurological diseases and aging that trigger not only neuronal degeneration but also changes in the mechanical properties of brain tissue. An understanding of the interconnected nature of the electro-chemo-mechanical processes that result in brain damage and ultimately loss of functionality is currently lacking. While modern mathematical models that focus on how to link brain mechanics to its biochemistry are essential in enhancing our understanding of brain science, the lack of experimental data required by these models as well as the complexity of the corresponding computations render these models hard to use in clinical applications. In this paper we propose a unified variational framework for the modeling of neuronal electromechanics. We introduce a constrained Lagrangian formulation that takes into account Newton's law of motion of a linear viscoelastic Kelvin–Voigt solid-state neuron as well as the classic Hodgkin–Huxley equations of the electronic neuron. The system of differential equations describing neuronal electromechanics is obtained by applying Hamilton's principle. Numerical simulations of possible damage dynamics in neurons will be presented.

## Introduction

Brain tissue is an inhomogeneous, multi-scale composite material composed of interconnected networks of blood vessels, neuron, and glia cells submerged in cerebrospinal fluid. Effects of mechanical and/or electro-chemical stresses and deformations on brain vary widely depending on the cell types, mechanical and bio-chemical characteristics of the cells, as well as cell's mechanosensitivity and mechanotransduction abilities. For instance, brain damage may take many different forms. For neurons, damage might include breakage of cytoskeleton networks in dendrites or axons, membrane rupture, separation of synaptic connections, or severance of dendritic or axonal projections. For the vascular system, damage might be puncture of macro or micro capillaries, or restrictions that alter perfusion on various scales. Depending on severity, most of these mechanical injuries will be followed by short or long term chemical imbalances and/or functional impairments or even death.

Given the high complexity of brain's structure and dynamics, designing, performing, and interpreting experiments on brain *in vivo* at various time and length scales continue to be very challenging and as a result the mechanisms that govern the interconnected electro-chemo-mechanical processes that result in brain damage and ultimately loss of functionality remain poorly understood. Mathematical models and corresponding computer simulations can increase our comprehension on brain damage processes (and, in general, on neurological diseases and neurodegeneration) and help design better experiments for measurements and hypothesis testing that ultimately will lead to improved medical diagnostic and therapeutic protocols. In the last few decades a multitude of mathematical models have been proposed to study brain biomechanics and, independently, brain bio-chemistry at cell as well as tissue levels. The majority of these models are mentioned in the comprehensive reviews of brain biomechanics and mechanobiology by Goldsmith (2001) and Goriely et al. (2015). Recently, models that link brain biomechanics to its bio-chemistry have also started to be developed (Drapaca and Fritz, 2012; Lang et al., 2015). Such coupled models are essential in enhancing the understanding of brain mechanisms such as the onset of normal pressure hydrocephalus due to ionic imbalances and in the absence of an elevated intracranial pressure (Drapaca and Fritz, 2012), and the propagation of damage in brain tissue caused by edema and lack of proper oxygenation (Lang et al., 2015). However, the lack of experimental data required by these very advanced mathematical models as well as the complexity of the corresponding computations render these models hard to use in today's clinical applications. In addition, these coupled models have been built at tissue level and thus they cannot predict the mechano-chemical responses of brain cells to mechanical and/or electro-chemical events that happen at tissue and organ scales.

The latest survey of the literature on brain biomechanics and mechanobiology by Goriely et al. (2015) emphasizes the current need in the field of brain research for the development of “*bottom-up”* mathematical models that link brain mechanics and function at each relevant length scale as well as across scales, incorporate anatomically accurate geometry and connections of cells and cerebral vasculature, and ultimately allow information from molecular and cellular levels to propagate to tissue and organ levels and *vice versa*. One possible first step in building such a bottom-up model is to start at the cell level and create an electromechanical model of neuronal dynamics. The aim of this paper is therefore to develop a lower-dimensional electromechanical model of a neuron which (1) is simple enough so that its predictions may be experimentally verified, and (2) could be used as a foundation model for more advanced multi-scaling (bottom-up) mathematical models. We assume that the electro-chemical activity of a neuron is described by the classic Hodgkin–Huxley equations (Hodgkin and Huxley, 1952) and that from a mechanical point of view the neuron behaves like a linear visco-elastic Kelvin–Voigt solid. The assumption of linear viscoelastic neuron is supported by experimental evidence reported by Lu et al. (2006) and Grevesse et al. (2015). In order to couple the Kelvin–Voigt mechanical model and the Hodgkin–Huxley electric model we will use a constraint Lagrangian formulation and the non-conservative form of Hamilton's principle. This approach will give us the coupled equations of motion by minimizing a special integral functional (action) whose integrand is made of kinetic and potential energies (Lagrangian) and the work done by the forces acting on the neuron. Although Hamilton's principle has been used in classical mechanics for a very long time (see for instance Lanczos, 1986), and recently has been applied to model neuronal electro-chemical activities (Dickel, 1989; Paninski, 2006; Wilson and Steyn-Ross, 2008; Chuankui, 2012) and ion transport through cell's membrane (Eisenberg et al., 2010), the principle has not been used to link neuron's mechanics and its electro-chemistry until now. The proposed electromechanical model has the following desirable features: (1) incorporates relevant macroscopic (cell level) and microscopic (ionic level) mechanical and electrical information, (2) facilitates the study of the dynamics of neuronal stiffness due to the evolutions of microstructural components, and (3) highlights neuronal mechanotransduction. We test the performance of our model in numerical simulations of neuronal mechanical insults. Although today it is well-known and accepted that traumatic brain injuries change the mechanics and electrophysiology of neurons on short and long time scales (see for instance Goriely et al., 2015, and the references within), the focus of the experimentalists as well as the modelers has been primarily on the mechanical characterization of the neuronal damage, and therefore a direct liaison between the neuronal mechanical properties and its altered functions has not been established yet. Our numerical simulations clearly show neuronal mechanotransduction: for initially applied displacements and speeds of magnitudes comparable to the size of the neuron, action potentials are observed, while very fast initially applied speeds (jabbing) inhibit the action potentials and this case might describe one possible neuronal damage dynamics following a serious mechanical injury. In addition, we notice that our proposed dynamics for the stiffness of a neuron appears to be in agreement with the experimental observations of healthy neurons reported by Zou et al. (2013).

The paper is organized as follows. In Section Mathematical Model we present our mathematical model, and in Section Results we show some relevant numerical simulations. The paper ends with a section containing concluding remarks and future directions.

## Mathematical model

**Figure 1:**
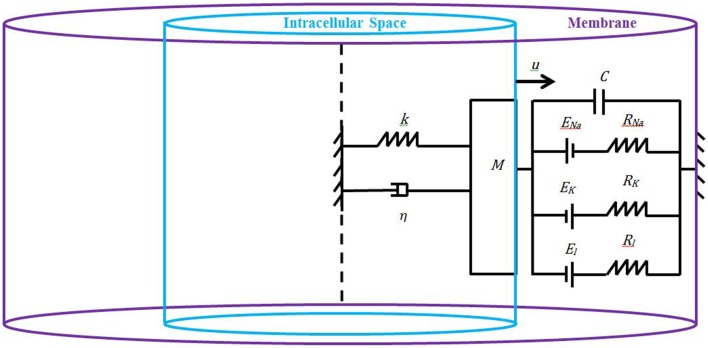
**Schematic of the proposed model: the neuron is an axi-symmetric homogeneous circular cylinder whose inner core is the intracellular space (light blue), and the outer layer is the membrane (purple)**. Due to the symmetry (dashpot line) and material homogeneity, it is enough to study half of the neuron whose properties are encapsulated into a spring-dashpot-mass mechanical system with the spring and dashpot connected in parallel (Kelvin–Voigt model), and the membrane is represented as an electric circuit governed by the classic Hodgkin–Huxley equations.

We model the axon as a axi-symmetric circular cylinder made of an inner core filled with the intracellular space and an outer layer filled with the cell's membrane (Figure 1). Both the intracellular space and the membrane are assumed to be homogeneous such that the study of neuronal electromechanics can be reduced to the study of a simple electromechanical element that we introduce here. Our novel low-dimensional electromechanical model of a neuron couples a spring-dashpot-mass mechanical model of the neuron and an electric circuit model of cell's membrane (Figure 1). Inspired by recent experimental findings by Lu et al. (2006) and Grevesse et al. (2015) we model the macroscopic material neuron as a linear visco-elastic Kelvin–Voigt solid. We use the classic Hodgkin–Huxley equations (Hodgkin and Huxley, 1952) to model the macroscopic electric dynamics of neuron's membrane. The linkage between the Kelvin–Voigt and Hodgkin–Huxley models is achieved by using a Lagrangian formulation and Hamilton's principle as follows.

We start by introducing a Lagrangian of the form:

(1) $$\begin{array}{l} ℒ=\frac{1}{2}M{\dot{u}}^{2}+\frac{1}{2}\tilde{a}{\dot{m}}^{2}+\frac{1}{2}\tilde{b}{\dot{n}}^{2}+\frac{1}{2}\tilde{c}{\dot{h}}^{2}-\frac{1}{2C\left(u\right)}q_{C}{}^{2}\\ \;-\frac{1}{2}k\left(m,n,h\right)\;u^{2}, \end{array}$$

where *M* is half of the constant mass of the neuron of constant cross-sectional area *A*, *u*(*t*) is the macroscopic (cell level) displacement that depends on time *t*, *k*(*m*,*n*,*h*) is the macroscopic spring constant (can be interpreted as a “rescaled” stiffness, as we will show in the results section), *C*(*u*) is the macroscopic capacitance of membrane's lipid bilayer modeled as a capacitor of electric charge *q*_*C*_, and $\tilde{a},\tilde{b},$ and $\tilde{c}$ are positive constants with physical units of Joules. Lastly, *m*(*t*),*n*(*t*), and *h*(*t*) are time-dependent variables between 0 and 1 representing the activations of the Na+ and K+ channels and, respectively, the inactivation of Na+ channel. For simplicity, we denote by $\dot{f}=\frac{df}{dt}$ the first order time derivative of a generic function *f*(*t*). The first term of the Lagrangian ℒ in Formula (1) is the macro-kinetic mechanical energy, while the second, third and fourth terms are micro-kinetic electric energies. The fifth term in Formula (1) represents a macro-potential electric energy and the last term of ℒ is a macro-potential mechanical energy.

Following the variational formulations for electric circuits (Ober-Blöbaum et al., 2013) and for neurons (Chuankui, 2012) we introduce *q*_*Na*_,*q*_*K*_, and *q*_*l*_, the electric charges of Na^+^, K^+^ and leakage channels, respectively. The law of charge conservation provides the following constraint:

(2) $$q_{C}+q_{Na}+q_{K}+q_{l}=0.$$

We take *q*_*Na*_, *q*_*K*_, *q*_*l*_, *m, n, h*, and *u* as generalized coordinates. By replacing *q*_*C*_ from Formula (2) into the Lagrangian expression (1) we can calculate the variation of the Lagrangian ℒ as follows:

(3) $$\begin{array}{l} \delta ℒ=lim_{\varepsilon \to 0}ℒ(q_{Na}+\varepsilon \delta q_{Na},q_{K}+\varepsilon \delta q_{K},q_{l}+\varepsilon \delta q_{l},m+\varepsilon \delta m,\\ \;n+\varepsilon \delta n,h+\varepsilon \delta h,u+\varepsilon \delta u)\\ \;=M\dot{u}\delta \dot{u}+\tilde{a}\dot{m}\delta \dot{m}+\tilde{b}\dot{n}\delta \dot{n}+\tilde{c}\dot{h}\delta \dot{h}+\frac{1}{C}q_{C}(\delta q_{Na}\\ \;+\delta q_{K}+\delta q_{l})+\frac{1}{2C^{2}}\frac{dC}{du}q_{C}{}^{2}\delta u-ku\delta u\\ \;-\frac{1}{2}\left(\frac{\partial k}{\partial m}u^{2}\delta m+\frac{\partial k}{\partial n}u^{2}\delta n+\frac{\partial k}{\partial h}u^{2}\delta h\right), \end{array}$$

where δ*q*_*Na*_, δ*q*_*K*_, δ*q*_*l*_, δ*m*, δ*n*, δ*h*, and δ*u* are variations of the generalized coordinates.

We further define the virtual work done by non-conservative forces as (Ober-Blöbaum et al., 2013; Cusumano et al., 2015):

(4) $$\begin{array}{l} \delta W=-(R_{Na}{\dot{q}}_{Na}\delta q_{Na}+R_{K}{\dot{q}}_{K}\delta q_{K}+R_{l}{\dot{q}}_{l}\delta q_{l}\\ \;+\eta \dot{u}\delta u)+(-E_{Na}\delta q_{Na}-E_{K}\delta q_{K}-E_{l}\delta q_{l}+F_{m}\delta m\\ \;+F_{n}\delta n+F_{h}\delta h+f\delta u) \end{array}$$

In Formula (4) the terms inside the first set of parentheses represent dissipative forces due to the resistors of resistances *R*_*Na*_,*R*_*K*_,*R*_*l*_ in the Hodgkin–Huxley model, and due to the linear dashpot in the Kelvin–Voigt model whose damping coefficient is η (can be interpreted as a “rescaled” dynamic viscosity, as we will show in the results section). The second set of parentheses in Formula (4) contains the following generalized forces: the reverse potentials *E*_*Na*_, *E*_*K*_, *E*_*l*_ of the Hodgkin–Huxley model, an externally applied mechanical force *f*, and forces *F*_*m*_, *F*_*n*_, *F*_*h*_ which are work conjugates of the gating variables *m*,*n*, and respectively *h*. The choice of signs in Formula (4) guarantees that the virtual work δ*W* is thermodynamically consistent.

We employ now the non-conservative form of Hamilton's principle:

(5) $$\int_{t_{1}}^{t_{2}}\left(\delta ℒ+\delta W\right)\;dt=0,$$

where the variations δ*q*_*Na*_, δ*q*_*K*_, δ*q*_*l*_, δ*m*, δ*n*, δ*h*, and δ*u* are independent and vanish at the arbitrary times *t*_1_, *t*_2_. By replacing Formulas (3) and (4) into the Hamilton's principle, Equation (5), using integration by parts, the independence of the variations δ*q*_*Na*_, δ*q*_*K*_, δ*q*_*l*_, δ*m*, δ*n*, δ*h*, δ*u* and the fact that these variations are zero at *t*_1_,*t*_2_, we obtain the following Euler-Lagrange differential equations:

(6) $$M\ddot{u}+\eta \dot{u}+ku-\frac{1}{2}\frac{dC}{du}V^{2}=f$$

(7) $$R_{Na}{\dot{q}}_{Na}=V-E_{Na}$$

(8) $$R_{K}{\dot{q}}_{K}=V-E_{K}$$

(9) $$R_{l}{\dot{q}}_{l}=V-E_{l}$$

(10) $$\tilde{a}\ddot{m}+\frac{1}{2}\frac{\partial k}{\partial m}u^{2}=F_{m}$$

(11) $$\tilde{b}\ddot{n}+\frac{1}{2}\frac{\partial k}{\partial n}u^{2}=F_{n}$$

(12) $$\tilde{c}\ddot{h}+\frac{1}{2}\frac{\partial k}{\partial h}u^{2}=F_{h}$$

where *V* = *q*_*C*_ ∕ *C* is the potential of the capacitor.

Lastly, Kirchhoff's current law needs to be added to the system of Equations (6–12) (Ober-Blöbaum et al., 2013). Replacing Equations (7–9) into Kirchhoff current law yields the well-known Hodgkin–Huxley equation for the membrane potential:

(13) $$C\dot{V}=I-\text{​​}\frac{1}{R_{Na}}\left(V-\text{​}E_{Na}\right)-\text{​}\frac{1}{R_{K}}\left(V-\text{​}E_{K}\right)-\frac{1}{R_{l}}\left(V-\text{​}E_{l}\right),$$

where *I* is a known external current applied on the membrane.

The unknown functions *u, V, m*,*n*, and *h* can be found by solving the coupled Equations (6, 10–13) with appropriate initial conditions. However, in order to solve these equations we need to provide expressions for $F_{m},\;F_{n},\;F_{h},\;\tilde{a},\;\tilde{b},\tilde{c},\;C(u),\;k(m,n,h)$. These expressions are very difficult to prescribe due to insufficient knowledge of neuronal mechanotransduction processes. Thus, for the sake of simplicity, we take *f* = 0 in Equation (6), and replace Equations (10–12) by the classic Hodgkin–Huxley equations for *m, n, h* (Dayan and Abbott, 2001):

$$\begin{array}{l} \dot{m}=\alpha_{m}\left(1-m\right)-\beta_{m}m\\ \dot{n}=\alpha_{n}\left(1-n\right)-\beta_{n}n\\ \dot{h}=\alpha_{h}\left(1-h\right)-\beta_{h}h \end{array}$$

where

$$\begin{array}{l} \alpha_{m}=\text{​ }\frac{0.1(V+40)}{1-exp\left(-0.1(V+40)\right)},\beta_{m}=4exp\left(-0.0556(V+65)\right),\\ \alpha_{n}=\text{​ }\frac{0.01(V+55)}{1-exp\left(-0.1(V+55)\right)},\beta_{n}=0.125exp\left(-0.0125(V\text{​}+\text{​}65)\right),\\ \alpha_{h}=\text{​ }0.07exp\left(-0.05(V+65)\right),\beta_{h}=\frac{1}{1+exp\left(-0.1(V+35)\right)}. \end{array}$$

In addition, we take (Dayan and Abbott, 2001):

(14) $$\frac{1}{R_{Na}}=g_{Na}m^{3}h\tilde{A},\frac{1}{R_{K}}=g_{K}n^{4}\tilde{A},\frac{1}{R_{l}}=g_{l}\tilde{A},$$

with Ã the surface area of the neuron, and *g*_*Na*_,*g*_*K*_,*g*_*l*_ the maximal conductances of the Na^+^, K^+^ and respectively leakage currents.

We propose further expressions for *C*(*u*) and *k*(*m*,*n*,*h*). According to Dayan and Abbott (2001) the capacitance is proportional to the surface area of the membrane and since our model is one-dimensional we could for instance assume that the membrane acts like a parallel-plate capacitor. Thus we have:

(15) $$C=c_{m}\tilde{A}=\frac{\epsilon \tilde{A}}{r+u}=\frac{\epsilon \tilde{A}}{r\left(1+u/r\right)}\approx \frac{\epsilon \tilde{A}}{r}\left(1-\frac{u}{r}\right),$$

where *c*_*m*_ is the specific membrane capacitance, ϵ is membrane's permittivity, and *r* is the thickness of the membrane. Regarding the expression for the dynamic spring constant *k*(*m*,*n*,*h*), we hypothesize that the cell stiffens during an action potential. Such an assumption appears to be supported by the observations made by Hille (2001) and Zou et al. (2013). During activation, the neuron stiffens due to the pulling on cytoskeletal elements by the swelling of the cell (Zou et al., 2013), and by the gates in ion channels that act as protein motors (Hille, 2001). According to Formula (14), Na^+^ conductance uses three gates of type *m* and one gate of type *h*, while K^+^ conductance uses four gates of type n and we could for instance assume that:

(16) $$k\left(m,n,h\right)=k_{0}\left(1+m^{3}(1-h)n^{4}\right),$$

where *k*_0_ is the spring constant in the inactive state of the neuron.

We observe that in the proposed model the electromechanical coupling is achieved through Equations (6, 10–12), and through Expressions (15–16).

## Results

In our simulations we used the following parameters taken from Dayan and Abbott (2001):

$$\begin{array}{l} E_{Na}=50mV,E_{K}=-77mV,E_{l}=-54.387mV,\\ g_{Na}=1.2\frac{mS}{mm^{2}},g_{K}=0.36\frac{mS}{mm^{2}},g_{l}=0.003\frac{mS}{mm^{2}}. \end{array}$$

The thickness of the membrane is *r* = 4 nm, the radius of the neuron is *r*_0_ = 2μ*m* (Dayan and Abbott, 2001), an average Young's modulus (stiffness) of the neuron is *E*_0_ = 200*Pa* (Lu et al., 2006; Zou et al., 2013), half of the neuronal mass is *M* = 0.1*ng* (Corbin et al., 2014). The specific membrane capacitance for a neuron in mechanical equilibrium (*u* = 0) is 0.01 $\frac{\mu F}{mm^{2}}$ and thus from Formula (15) we have:

$$c_{m}=0.01\left(1-\frac{u}{r}\right)\frac{\mu F}{mm^{2}}.$$

We also used a value of μ = 4*mPa*·*s* for the dynamic viscosity of the neuron (this value was found by Park et al. (2010) for non-neuronal cells).

Under the assumption that the neuron has a circular cylindrical shape, the cross-sectional area is $A=\pi r_{0}{}^{2}$. Then the spring constant of an inactive neuron is calculated from equating two different representations of the restoring force in a linear elastic spring: $k_{0}u=E_{0}\frac{u}{r_{0}}A$. Thus $k_{0}=E_{0}\frac{A}{r_{0}}$. Similarly, the damping constant is calculated from the shear force to be: $\eta =\mu \frac{A}{r_{0}}$. Lastly, in all numerical simulations we applied a constant external current per unit (surface) area of 0.1$\frac{\mu A}{mm^{2}}$.

Because of the numerical stiffness of Equation (6) we solved instead $M\ddot{u}+\eta \dot{u}+ku=0$, with *k* given by Formula (16), and we solved the classic Hodgkin–Huxley equations with *C* given by Formula (15). We notice that this simplification preserves a weaker coupling between the mechanical and electrical behaviors of the neuron expressed by Formulas (15) and (16).

We re-wrote $M\ddot{u}+\eta \dot{u}+ku=0$ as a system of first order differential equations:

(17) $$\dot{u}=d,\dot{d}=-\frac{\eta }{M}d-\frac{k}{M}u$$

and used Matlab built-in function **ode15s** that solves stiff ordinary differential equations. The function **ode15s** uses a modified linear multistep backward difference formula of order up to five known to have good stability and changes the stepsize of the discretization adaptively according to a numerical scheme that calculates relative and absolute error tolerances (Shampine and Reichelt, 1997).

The Hodgkin–Huxley equations were solved with the following initial conditions:

$$\begin{array}{l} V\left(0\right)=-65mV,m\left(0\right)=\frac{\alpha_{m}\left(V(0)\right)}{\alpha_{m}\left(V(0)\right)+\beta_{m}\left(V(0)\right)},\\ n\left(0\right)=\frac{\alpha_{n}(V\left(0\right))}{\alpha_{n}\left(V(0)\right)+\beta_{n}(V(0))},h\left(0\right)=\frac{\alpha_{h}(V(0))}{\alpha_{h}\left(V(0)\right)+\beta_{h}(V(0))}. \end{array}$$

We solved System (17) using two sets of initial conditions:

$$\begin{array}{l} \text{Set}1:u\left(0\right)=1nm,d\left(0\right)=\dot{u}(0)=10nm/ms\\ \text{Set}2:u\left(0\right)=0,d\left(0\right)=\dot{u}(0)=1nm/\mu s \end{array}$$

**Figure 2:**
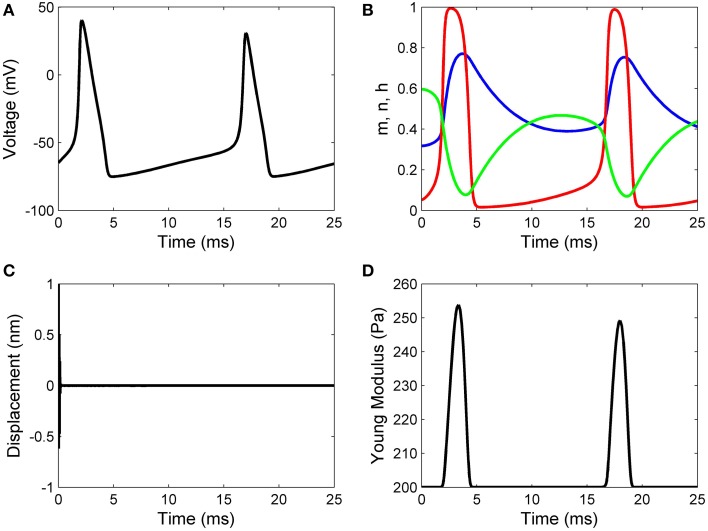
**Results for initial displacement of 1 nm and initial speed of 10 nm/ms: (A) voltage, (B) functions n (blue line), m (red line), and h (green line), (C) displacement, and (D) Young's modulus**.

**Figure 3:**
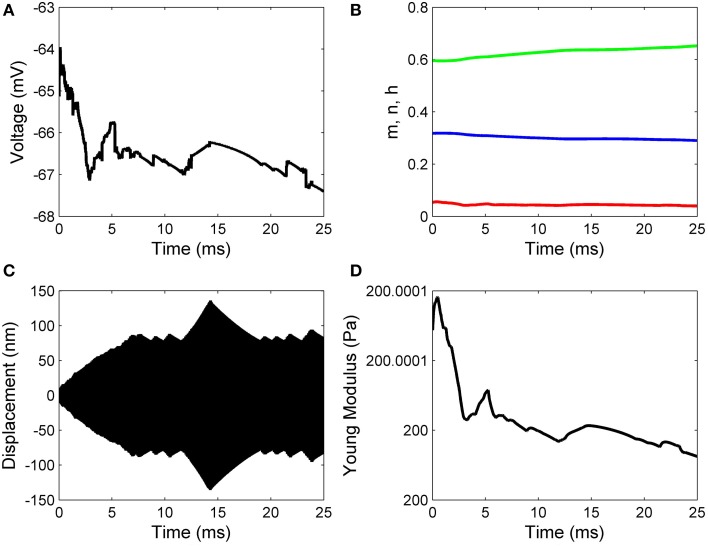
**Results for zero initial displacement and initial speed of 1 nm/μs: (A) voltage, (B) functions n (blue line), m (red line), and h (green line), (C) displacement, and (D) Young's modulus**.

**Figure 4:**
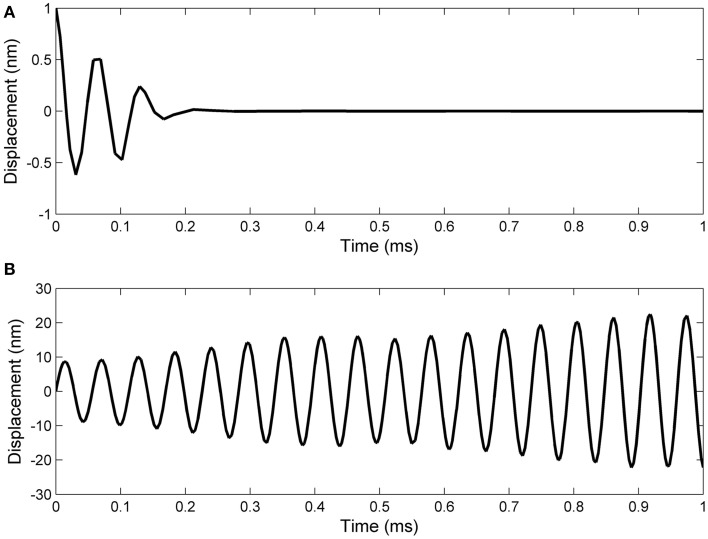
**A closer look at the displacement evolution during 1 ms in the following cases: (A) initial displacement of 1 nm and initial speed of 10 nm/ms; (B) zero initial displacement and initial speed of 1 nm/μs**.

Working with Matlab's default values for the relative error tolerance (10^−3^) and the absolute error tolerance (10^−6^), the function **ode15s** solved System (17) and the classic Hodgkin–Huxley equations coupled by Formulas (15) and (16) using a minimum (maximum) stepsize of 0.0051 *ms* (1.0076*ms*) for the initial conditions in Set 1, and a minimum (maximum) stepsize of 0.00075 *ms*(0.0067*ms*) for the initial conditions in Set 2. In Figures 2, 3 we show the evolutions of the voltage, gaiting variables, displacement and Young's modulus for Set 1 respectively, Set 2. For initial conditions in Set 1, we observe that the action potentials occur and the Young's modulus and the displacement variations appear to be physically admissible and possibly within a healthy range. The dynamics of the stiffness of a neuron is in agreement with the experimental observations in the normally functioning regime reported by Zou et al. (2013). However, for the initial conditions in Set 2 which mimic a more serious traumatic event, not only that the action potentials do not happen anymore (Figure 3A) but also sustained big oscillations of the displacement field are noticed (Figure 3C). In this case the gaiting variables (Figure 3B) as well as the Young's modulus (Figure 3D) remain almost constant. The solutions obtained using the initial conditions in Set 2 might show damaging effects of a very fast initial speed (jabbing) on the material structure and electro-chemical activity of a neuron. As it is apparent from Figures 2, 3, the proposed model is able to capture neuronal mechanotransduction. In Figure 4 we show the evolutions of the displacements obtained using the two sets of initial conditions during 1 ms. While the oscillations are quickly attenuated for Set 1 of initial conditions (Figure 4A) which allows the action potential to develop soon afterwards, for the initial conditions of Set 2 the amplitudes of the oscillations of the displacement field are much higher than in the previous case and do not appear to diminish in time. Also, the membrane's depolarization does not occur in this case (Figure 4B).

## Conclusions and future directions

In this paper we proposed a new electromechanical model that couples the mechanical behavior and electro-chemical activity of a neuron and investigated neuronal mechanotranduction through numerical simulations. The neuron was modeled as a liner-viscoelastic Kelvin–Voigt solid whose electro-chemical activity was described by the classic Hodgkin–Huxley equations. We used a Lagrangian formulation and Hamilton's principle to obtain the coupled equations of motion. This approach has the advantage that it can link macroscopic (cell level) as well as microscopic (ionic level) mechanical and electrical information and thus it can describe neuronal mechanotransduction. In addition we assumed that the membrane's capacitance depends on the mechanical displacement of the neuron, while the Young's modulus of the neuron depends on the gating variables in the Hodgkin–Huxley model. Our numerical simulations were done in Matlab using the built-in function **ode15s** to solve a simplified version of our differential equations. When a constant external electric current was applied and the initial displacement and speed were of orders of magnitude comparable to the size of the neuron, the action potentials occurred and looked similar to the ones observed in healthy neurons. In this case the dynamics of the neuron's stiffness appeared to be in agreement with experimental measurements done on healthy neurons (Zou et al., 2013). However, for very fast initial speeds which could model a serious traumatic event and in the presence of a constant applied external current, high persisting oscillations in the mechanical displacement of the neuron were observed and the action potentials did not happen, suggesting possible structural and functional damage of the neuron.

One of the limitations of the proposed model is coming up with physically relevant expressions that couple capacitance and displacement and respectively stiffness and gating variables, because there are no experimental observations that could guide us. However, given the simplicity of the proposed model, we hope that our approach will inspire future experimental work that will provide empirical relationships among the model's mechanical and electrical parameters. Another limitation of our approach is the use of the Matlab built-in function **ode15s** to solve the proposed system of stiff differential equations. Shampine and Bogacki (1989) advised caution in drastically reducing the stepsize in the discretization implemented in **ode15s** since this action may in fact increase numerical error and cause instabilities in the solutions.

In our future work we plan to develop a better numerical solver that will allow us to solve the fully coupled differential equations which are numerically stiff. In addition, we will perform the bifurcation analysis of the model. Lastly, we intend to incorporate in our model ion transport through cell's membrane using the variational formulation from Eisenberg et al. (2010).

## Conflict of interest statement

The author declares that the research was conducted in the absence of any commercial or financial relationships that could be construed as a potential conflict of interest.
