# Corrigendum: An electromechanical model of neuronal dynamics using Hamilton's principle

**DOI:** 10.3389/fncel.2015.00339

**Published:** 2015-08-28

**Authors:** Corina S. Drapaca

**Affiliations:** Department of Engineering Science and Mechanics, Pennsylvania State University, University Park PA, USA

**Keywords:** electromechanics, dynamicstiffness, Kelvin–Voightmodel, Hodgkin–Huxleymodel, Hamilton's principle

In Equation (3) the limit $lim_{\varepsilon \;\to \;0}L$ should be replaced by $lim_{\varepsilon \to 0}\;dL/d\varepsilon .$

## Conflict of interest statement

The author declares that the research was conducted in the absence of any commercial or financial relationships that could be construed as a potential conflict of interest.

